# Effect of Lavage Solution Type on Bronchoalveolar Lavage Fluid Cytology in Clinically Healthy Horses

**DOI:** 10.3390/ani13162637

**Published:** 2023-08-15

**Authors:** Cornélie M. Westermann, Annelieke G. de Bie, Carla Olave, Janny C. de Grauw, Erik Teske, Laurent L. Couetil

**Affiliations:** 1 Department of Clinical Sciences, Faculty of Veterinary Medicine, Utrecht University, Yalelaan 114, 3584 CM Utrecht, The Netherlands; 2 Dierenkliniek de Vijfsprong, Vijfsprongweg 28, 6733 JJ Wekerom, The Netherlands; 3 Department of Veterinary Clinical Sciences, College of Veterinary Medicine, Purdue University, West Lafayette, IN 47907, USA; 4 Department of Clinical Sciences and Services, Royal Veterinary College, London AL9 7TA, UK

**Keywords:** lung lobes, equine, saline, phosphate-buffered saline (PBS), Ringer’s, Plasma-Lyte

## Abstract

**Simple Summary:**

One of the most valuable methods to diagnose lower airway disorders such as equine asthma is broncho alveolar lavage (BAL), whereby a solution, usually saline, is inserted in a distal part of a lung lobe. The solution is then collected and contains cells present in the deeper airways. It is a safe procedure, but saline can cause local inflammation (neutrophilia). This study aimed to investigate if there are better solutions for the procedure, for example, less acidic ones, to avoid causing inflammation, and whether the BAL fluid retrieved from one lung lobe is representative for the whole lung. Since it is not necessary to use horses with respiratory disorders to answer these research questions, healthy horses were used. Four horses were used (using four lung lobe locations and four different solutions per horse twice with an interval of 48 h) and generated enough data to answer the questions. The findings were that the BAL composition is not affected by either the solution used or the lung lobe sampled. These findings are positive because they support keeping the procedure easy and practical.

**Abstract:**

Equine bronchoalveolar lavage (BAL) is usually performed with 250–500 mL of isotonic saline at pH 5.5. The acidic pH of saline may cause an increase in airway neutrophil count 48 h after BAL. Other isotonic solutions such as Ringer’s solution, phosphate-buffered saline (PBS) or Plasma-Lyte 148^®^ have a neutral pH of 7.4 and might be a better choice for BAL by not provoking inflammation and the influx of neutrophils into airways. BAL was performed in four healthy horses in four different lung lobes using four different solutions in a randomized crossover design. In each lobe, BAL was performed twice with a 48 h interval using 250 mL of solution. Automated total nucleated cell counts (TNCs) were recorded, and differential cell counts in lavage fluid were determined by two investigators blinded to treatments. The mean volume of BAL fluid retrieved was 51 ± 14%. The mean neutrophil percentage (%N) increased from 1.5 ± 0.9% to 14.7 ± 9.6% at 48 h (*p* < 0.001) but was not significantly affected by the solution used or the lung lobe sampled. In conclusion, in this study, the influx of neutrophils into airways after BAL was independent of the type of isotonic solution used and the lung lobe sampled. Saline remains an appropriate solution for BAL in horses.

## 1. Introduction

The diagnosis of equine asthma (EA) is often based on a combination of clinical findings and diagnostic tools, such as bronchoalveolar lavage (BAL), endoscopic examination, and lung function tests [1,2,3,4]. Affected horses have excessive tracheal mucus, mild-to-severe airflow limitation during disease exacerbation, and an increase in the number of inflammatory cells in the BAL fluid compared with reference values (neutrophils ≤ 5%, eosinophils ≤ 1%, and mast cells ≤ 2%) [1]. BAL can be performed during bronchoscopic examination of the lower airways or by using a cuffed BAL catheter [1,5]. The procedure does not significantly disturb the lung function of healthy horses [6], although Sweeney et al. (1994) found that the neutrophil count was elevated 48 h after BAL [7]. One study even showed an improvement in airway function after BAL, which was attributed to the removal of mucus [8].

The BAL procedure is most commonly performed by infusing 250–500 mL of sterile, warm (37 °C) 0.9% saline [5,6,9]. However, the endobronchial instillation of sterile isotonic saline results in significant airway neutrophilia of similar magnitude in both healthy and asthmatic horses 5 h later [10]. Saline is isotonic but has a pH of around 5.4–5.5 and might be irritating to the airways [11]. Although the pH of the pulmonary epithelial lining is lower than that of the body, little is known about how airways respond to lavage solutions of different pH values [12].

In cats, dogs, and humans, as in horses, sterile isotonic saline (warmed to body temperature) is routinely used for BAL [11,13,14]. In swine and cattle, BAL is commonly performed using phosphate-buffered saline (PBS) [15,16]. Indeed, other isotonic solutions such as Ringer’s solution, PBS, or Plasma-Lyte 148^®^ have a pH of 7.4 and might be a better choice for BAL in horses.

The main objective of this study was to compare BAL fluid cytology 48 h after BAL with four different types of solutions (saline, Ringer’s solution, PBS, and Plasma-Lyte 148^®^) in clinically healthy horses. Our hypothesis was that saline, as a BAL solution, induces a greater inflammatory reaction than Ringer’s solution, PBS, or Plasma-Lyte. If this is the case, other isotonic fluids with a neutral pH should preferentially be used for this procedure.

The second aim of the study was to evaluate possible differences in BAL cytology between the four different lung lobes sampled during this study. Our underlying hypothesis was that there would be no difference in the cytology between the lung lobes and that the cytological response to lavage fluid would be the same regardless of the lung lobe sampled.

## 2. Materials and Methods

### 2.1. Horses

This randomized crossover study used four healthy Dutch Warmblood mares, owned by the Utrecht University Equine Clinic (Utrecht, the Netherlands). The horses were between 7 and 11 years old and weighed 614.75 kg ± 32.9 at first sampling. The horses were individually housed in identical low-dust environments (dust-free shavings or at pasture) and were fed low-dust feed (haylage and pellets). They were clinically healthy and free of respiratory signs, as assessed with two clinical scores [17,18], and had not been treated with medication for at least two weeks before the start of the study. The horses were exercised with a horse walker for an hour daily, except for the day before, the day of, and the day after BAL. The Institutional Animal Ethics Committee of the Utrecht University Faculty of Veterinary Medicine, The Netherlands, approved the experiment (AVD1080020185204).

### 2.2. Study Design

Bronchoalveolar lavage (BAL) was performed during weeks 1, 3, 5, and 7. The first BAL (baseline) was performed on a Monday with two horses, followed by a second, identical procedure 48 h later (same solution per lung lobe and exactly the same location within the lung). The other two horses had their first BAL on Tuesday, again with a second identical BAL 48 h later. After the second BAL, all horses had a 12-day washout period. The horses were examined clinically every day, from two days before the first BAL until two days after the second BAL. Two previously reported clinical scoring systems were used: the clinical score of Tesarowski et al. (‘long score’), ranging from 0 to 21, which is based on respiratory rate, nasal discharge, respiratory effort, tracheal sounds, crackles, wheezes, cough, and abdominal effort on exhalation [17], as well as the clinical score of Rush et al. (‘short score’), ranging from 2 to 8, focusing on nostril flaring and abdominal effort [18]. In both scoring systems, a higher score indicates more severe clinical disease. Clinical scores were assigned by one observer who was blinded to the treatment allocation.

### 2.3. BAL

**Figure 1:**
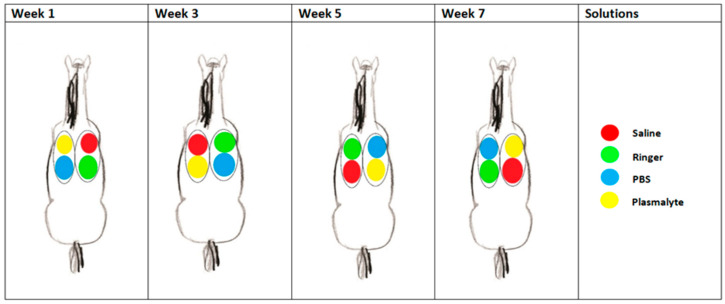
Scheme of one of the horses during the study, showing which fluid was instilled at each location per sampling time point.

Four different lung lobes were sampled, always in the same order, using each of the four types of solution, according to a randomized, crossover design (Figure 1). The lung lobes sampled at each time point in each horse were the right caudodorsal lobe (RCaL), the right accessory lobe (RAcL), the left caudodorsal lobe (LCaL), and the left cranial lobe (LCrL), as described by Smith et al. [19]. In total, 250 mL of 0.9% saline “Manufacturer Braun (Melsungen, Germany)”, Plasma-Lyte 148^®^ “Manufacturer Baxter Healthcare Corporation (Deerfield, IL, USA)”, Ringer’s solution “Manufacturer Braun (Melsungen, Germany)”, and PBS “Manufacturer Sigma-Aldrich Chemie GmbH (Steinheim, Germany)” at room temperature was instilled in each lung lobe. Each solution was given a color code (red for saline, green for Ringers, blue for PBS, and yellow for Plasma-Lyte 148^®^) (Figure 1). Each horse received a different type of solution in the same lung lobe every other week. Schedules were allocated randomly to the horses through a ballot draw.

After the horse was sedated with detomidine “Manufacturer Orion Corporation (Espoo, Finland)” (0.01 mg/kg IV) and butorphanol “Manufacturer Intervet (Boxmeer, The Netherlands)” (0.02 mg/kg IV) and a twitch was applied, a bronchoscope “Manufacturer Endotechniek BV (Leiderdorp, The Netherlands)”, (2.5 m length, 9 mm diameter), was passed through the right ventral meatus into the nasopharynx, after which the trachea was entered, holding the endoscope in the middle of the trachea until the bifurcation was reached [20]. Lidocaine hydrochloride “Manufacturer Richter Pharma (Wels, Austria)” (2%, pH 5.5), measured with an Inolab pH device “Manufacturer GmbH & Co (Weilheim, Germany)” was sprayed (20 mL/horse) onto the carina and into the bronchus to be sampled. The endoscope was advanced into the widest generation bronchus in the correct lung lobe until wedged. The BAL solution (250 mL) was manually instilled through the endoscope biopsy channel into the lobe with 5 × 50 mL syringes, immediately followed by manual aspiration using the same syringes. The first 20 mL of the retrieved BAL solution was discarded because this corresponded to the volume of the endoscope biopsy channel and therefore had not reached the airways. The remaining BAL fluid was filtered through a gauze and immediately put on ice. The volume of fluid retrieved was measured. The procedure was repeated for the other three lung lobes. All samples were sent to the laboratory on ice and were processed within half an hour of collection.

### 2.4. Cell Counts and Cytological Evaluation

The total cell count was measured with an automated hematology analyzer (ADVIA 2120i^®^) “Manufacturer Siemens Healthcare Nederland BV (Den Haag, The Netherlands)”, using the Basophil/Lobularity and RBC/Plt channels for the total nuclear cell (TNC) count and red blood cell (RBC) count, respectively.

Cytospin was performed [Thermo Scientific™, “Manufacturer Thermo Fisher Scientific (Waltham MA, USA)”] at 650 RPM for 10 min. Two different slides were prepared in duplicate using 120 µL and 280 µL of the samples. After air drying, the slides were stained with May Grünwald Giemsa stain.

Two of the authors (CW and CO), blinded to treatment allocation, counted 300 cells in duplicate per lobe per sampling date using a similar Z-pattern for all slides. The average counts were used. Cytological analysis of the samples included TNC count, erythrocyte count, and differential leukocyte count, which are expressed as percentages, including macrophages, lymphocytes, neutrophils, eosinophils, and mast cells. Other findings, such as fibers and/or ciliated epithelial cells were recorded.

### 2.5. Statistical Analysis

Sample size calculations were performed prior to ethical approval with the help of a statistician and an online calculator (http://ausvet.com.au: resources > epitools > sample size calculation, accessed on 14 November 2018). Given an estimated increase in the neutrophil count in saline BAL fluid between baseline and 48 h of 32.6% [7], an estimated increase of only half this magnitude (16.3%) for the other three BAL fluids, a power of 80%, an alpha value of 0.05 (Bonferroni adjusted), and a one-sided *t*-test, four horses would be needed for each fluid.

Statistical analyses were performed using the statistical software package SPSS 26.0 “Manufacturer IBM Corp. (Armonk, NY, USA)”.

The presence of a main effect of BAL solution type or lung lobe on BAL cytology after 48 h was verified for all solutions and all lobes with a type III test for fixed effects. The ANOVA F-test was used to determine the effect of each solution on BAL fluid cytology.

The effect of time (week) and any possible influence of repeated BAL on changes in BAL cytology over time, as well as the significance of any time–BAL solution interactions, were tested with a linear mixed model for repeated measures with horse as a random (subject) effect, and time (week), lobe, and BAL–solution type as fixed effects and repeated factors.

The normality of data was assessed using Kolmogorov–Smirnov test, and Bonferroni’s correction was used to adjust for multiple comparisons. Neutrophil percentage, mast cell percentage, and total cell counts were log-transformed to achieve a normal distribution. The level of significance was set at *p* < 0.05 for all statistical analyses.

Data are presented as mean ± SD.

## 3. Results

### 3.1. General

The dataset of all four horses consisted of 32 BAL samples per week (four horses × four lung lobes × two time points), with two duplicate cytospin slides for each BAL, giving a total of 128 BAL samples and 256 cytospins and cell counts for the entire study (Supplementary Materials). No samples were missing or excluded from analysis.

None of the horses showed any signs of discomfort or clinical disturbance in lung function as a result of the procedure, as evidenced by a mean Tesarowski clinical score over the entire study period of 0.42 (range 0–2) out of 21 points, and a mean Rush score of 0.0 (range 0–0) out of 8 points.

The mean return after manual aspiration was 127.7 ± 34.0 mL (51.1 ± 13.6% recovered BAL fluid). Erythrocytes were not detected in any sample by the ADVIA 2120i or by the cytologists.

### 3.2. Effect of BAL Solution Type on Response to BAL Procedure

**Figure 2:**
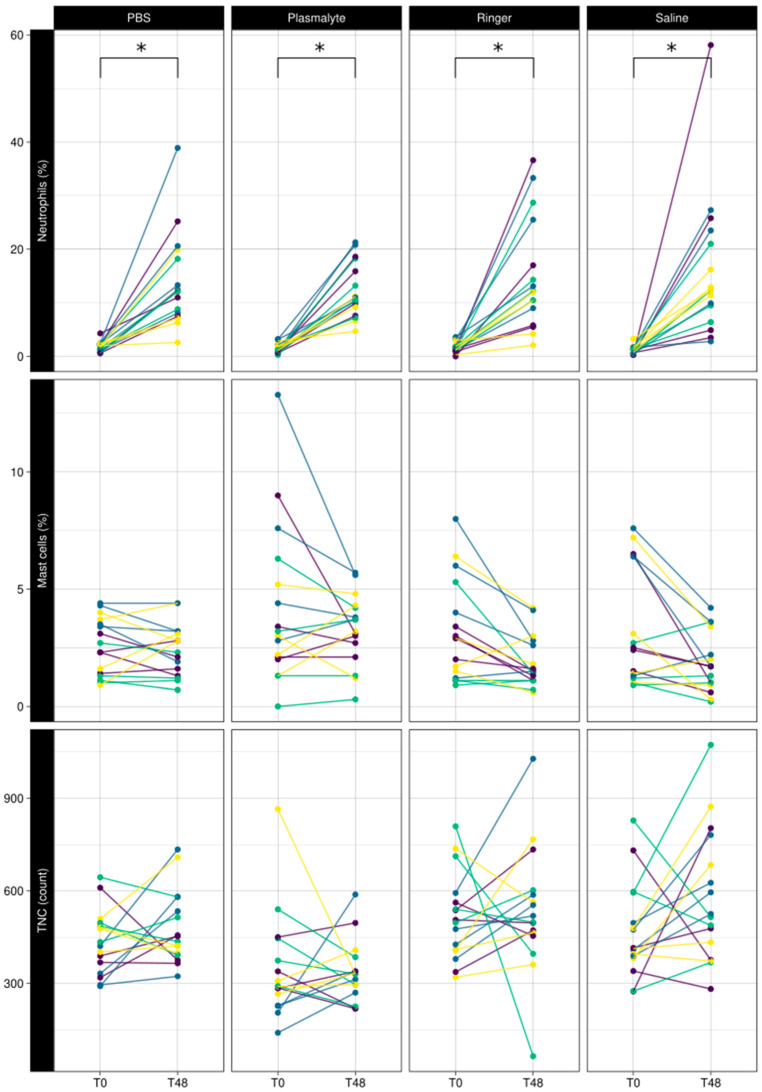
Comparison of baseline (T0) vs. 48 h (T48) of neutrophil percentage, mast cell percentage, and total nucleated cell counts in BAL fluid using different lavage solutions. Each color represents a horse. Lines connect 2 measurements in the same lung lobe. * Significant difference.

The mean neutrophil percentage increased from 1.5 ± 0.9% to 14.7 ± 9.6% 48 h after BAL (*p* < 0.001) regardless of the BAL solution used (Figure 2), while TNC and mast cell percentages were not affected by BAL.

### 3.3. Effect of Lung Lobe (Sampling Site) on Response to BAL Procedure

**Figure 3:**
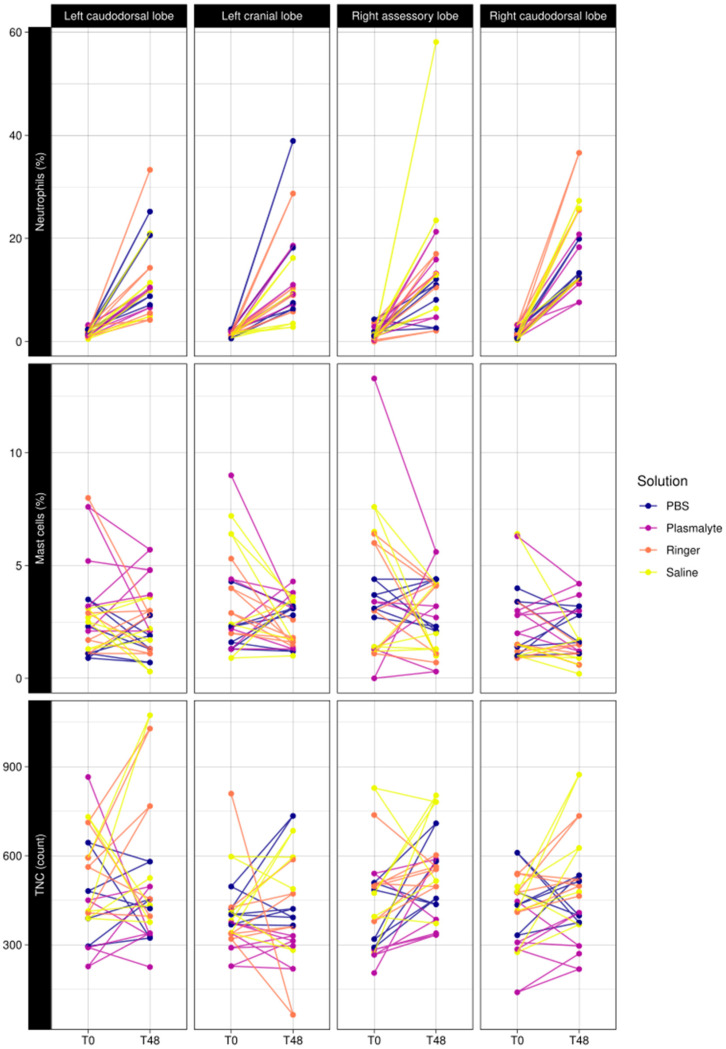
Comparison of baseline (T0) vs. 48 h (T48) of neutrophil percentage, mast cell percentage, and total nucleated cell counts in BAL fluid in the different lung lobes (left caudodorsal lobe (LCaL), left cranial lobe (LCrL), right accessory lobe (RacL), and right caudodorsal lobe (RCaL).

TNC, neutrophil percentage, and mast cell percentage were not significantly different in the different lung lobes sampled in either the first or second BAL procedures (Figure 3).

### 3.4. Effect of Time

**Figure 4:**
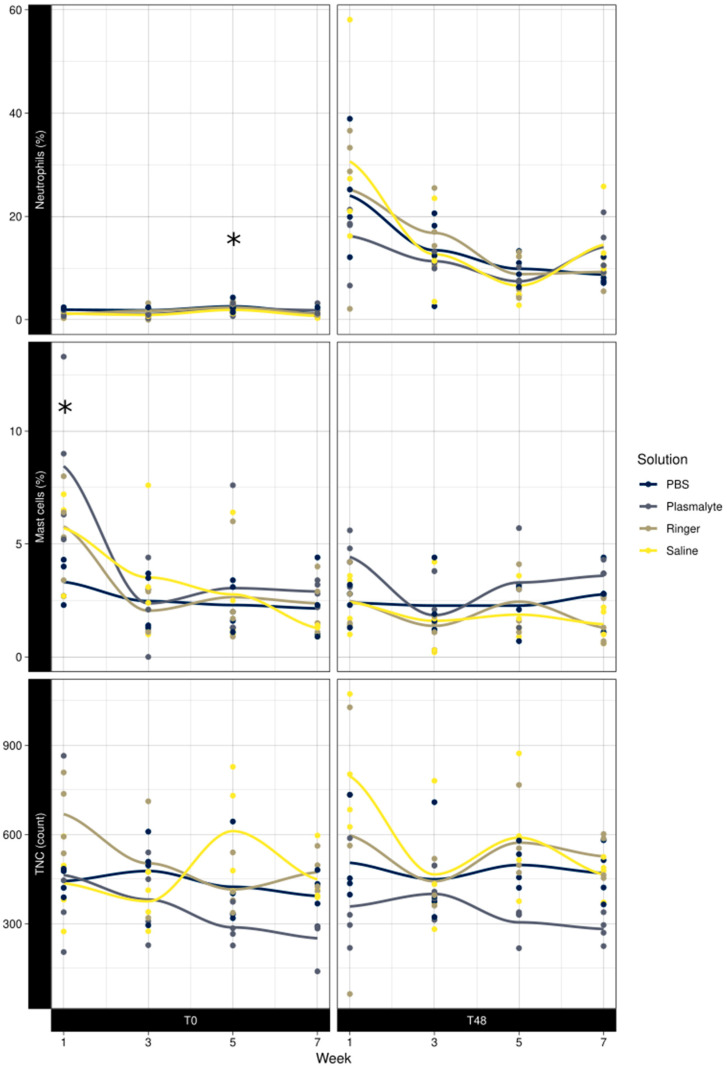
Effect of week at T0 and T48 on neutrophil percentage, mast cell percentage, and total nucleated cell counts in BAL fluid. * Significant difference.

There was no significant difference in the baseline TNC at any of the time points studied. The baseline neutrophil percentage was significantly higher in week 5 than in weeks 1, 3, and 7 (*p* = 0.01); the mast cell percentage was significantly higher in week 1 than in weeks 3, 5, and 7 (*p* < 0.001) (Figure 4).

## 4. Discussion

To the best of our knowledge, this is the first study in horses to evaluate the effects of saline and three other solutions on the total and differential cell count in BAL fluid. All solutions used for BAL, regardless of pH, resulted in a statistically significant increase in neutrophil percentage 48 h after the procedure but not in a statistically significant increase in the total number of nucleated cells. The increase in neutrophils was clinically relevant, being indicative of mild-to-moderate equine asthma in diagnostic BAL procedures [1]. No significant differences between the solutions or sampling locations were found. Thus, our hypothesis that a more acidic solution (0.9% saline) would result in a more pronounced influx of inflammatory cells in lavaged airways at 48 h compared with solutions with a neutral pH was rejected. However, our second hypothesis was confirmed—there were no differences in cytology between lung lobes, neither at baseline nor in the response to the lavage solution used.

### 4.1. Influx of Inflammatory Cells

The neutrophil percentage increased from 1.5 ± 0.9% to 14.7 ± 9.6% at 48 h (*p* < 0.001) after the first BAL. Our findings are similar to those of Sweeney et al. (1994), who reported a statistically significant increase in absolute neutrophil cell count (from 13 to 160 cells/μL, *p* = 0.01) and neutrophil percentage (from 4.8% to 40.6%, *p* = 0.002) 48 h after infusion of 100 mL of sterile saline (pH = 5.7) [7]. The variance observed was in line with that seen in previous studies and may be inherent to multiple sampling [6].

McGorum et al. (1993) found that instillation of even a small volume (5 mL) of neutral pH PBS in horses’ airways resulted in neutrophilic airway inflammation detected by BAL performed 5 h later [10]. Unfortunately, the effect of such instillation at later time points is not known.

In humans, the instillation of 10 mL of 0.9% saline solution in a lung segment resulted in a mild increase in the proportion of neutrophils in BAL fluid that peaked at 24 h (10 ± 8%) and returned to baseline values at 48 h (5 ± 2%) [21]. The exposure of the lower airways to acidic fluids as a result of gastric reflux in humans or experimental instillation in animal models results in an influx of inflammatory cells, in particular neutrophils [22].

In the current study, a possible effect of 2% lidocaine on the BAL fluid pH cannot be ignored; however, less than 5 mL was used, relative to the 250 mL of BAL solution. The average proportion of pulmonary epithelial lining fluid collected after BAL represents 0.79% and 6.43% of the BAL solution instilled, depending on whether the measurement was based on the urea or inulin method, respectively [23]. If we assume the pH of the pulmonary epithelial lining of horses to be 6.6, we calculated that the change in BAL fluid pH caused by the 2% lidocaine solution would be negligible compared with the change in pH due to the BAL solution used (<2.2%).

Both neutral and acidic pH solutions induced neutrophilic airway inflammation at 48 h after instillation, suggesting that other factors besides pH are at play. Lung lavage with a large volume of saline solution is an established method to deplete airway surfactant and induce acute lung injury in rodent models [24]. The collection of BAL fluid in healthy horses usually yields a thick layer of foamy fluid rich in surfactant proteins [25]. Therefore, it is possible that the neutrophilic influx in horses’ airways 48 h after the BAL procedure was the result of localized surfactant depletion. After a 12-day washout period, the neutrophil percentage had returned to 1.53 ± 0.90%, suggesting that the 12-day washout period was appropriate for this study. Despite the marked neutrophilic inflammation, no respiratory or systemic clinical signs were detected, suggesting that the localized airway inflammation was subclinical.

### 4.2. Lung Lobes

In the present study, both lungs were sampled in two different locations. There was no differences in the neutrophil or mast cell percentages between the left and right lungs. The percentage of mast cells at baseline tended to be higher, but not significantly so, in the right accessory lobe than in the other lobes. Potential differences in relative cell counts between the left and right lungs have been studied before, with conflicting results [26,27,28]. Depecker et al. (2014) found the right lung to have a higher neutrophil percentage than the left lung (10% versus 9%) in racehorses [28]. It was speculated that the difference was possibly due to the straighter disposition of the right main stem bronchus, which is responsible for a greater exposure of the right lung to allergens and (micro-)organisms, thereby eliciting a neutrophil response [28]. Sweeney et al. (1992) found no significant difference in neutrophil percentage between the left and right lung lobes, but a difference in mast cell percentage, with more mast cells being found in the left lung [27]. Jean et al. (2011) reported a difference in the mast cell numbers between the left and right lungs (right lung 1.74 ±0.32% and left lung 1.18 ± 0.21%) [26].

It is hard to relate our findings to these data given the inconsistencies in the previously reported results. Overall, and bearing in mind that this study employed a limited number of healthy horses, it would appear that lung sampling location does not greatly affect BAL fluid cytology relative to other factors (e.g., time after fluid instillation).

### 4.3. Factors in the BAL Procedure

#### 4.3.1. Return Volume

During the study, a BAL return volume of 51.1% was achieved. Jean et al. (2011), using two incremental instillations of 250 mL of isotonic saline (0.9% NaCl) solution in the same bronchial site, retrieved a significantly lower volume of BAL fluid from horses with severe equine asthma (16.6%) than from healthy horses (36.7%) [26]. Another study, using different BAL volumes (50, 3 × 100, and 300 mL), reported recovery of BAL fluid of 50–60% from healthy horses (closely mirroring our average return), while retrieving 24% from horses with severe equine asthma [27].

#### 4.3.2. Volume Used

A total volume of 250–500 mL of solution is usually recommended for performing BAL [5,29]. Since four sample locations were used in each session, the lower limit of the advised volume (250–500 mL) in the consensus statement was used per location [1]. Orard et al. (2016) studied the influence of BAL volume (250 mL versus 500 mL of saline) on cytological profiles, finding a lower total cell count and neutrophil percentage with the larger volume [29]. There was poor agreement between the findings when 250 mL or 500 mL of BAL solution was used for diagnosing inflammatory airway disease, with a similar proportion of horses being classified as healthy or having inflammatory airway disease [30]. Sweeney et al. (1992) reported a higher percentage of neutrophils and a lower percentage of mast cells when a smaller volume (50 mL) was used compared with a larger volume (350 mL) [27].

#### 4.3.3. Temperature of the Fluid

Although it is recommended to use BAL solutions at 37 °C, this was not possible given the study design, so we used BAL solutions at room temperature [4]. Further research is necessary to determine whether warmed (37 °C) solutions have different outcomes compared with solutions used at room temperature.

#### 4.3.4. Healthy Horses Versus Asthmatic Horses

Healthy horses were used in this study. While asthmatic horses may have a different reaction to the BAL procedure, for instance, regarding relative increase in neutrophils, it is arguably less likely that cell count results would differ by lung lobe sampled or solution type used. Moreover, asthmatic horses cough and have dyspnea, which can complicate the procedure and might have hindered drawing any conclusions on the effect of solution type and sampling site per se.

#### 4.3.5. Multiple BAL Procedures over Time

Although the neutrophil percentage was significantly higher in all horses at week 5 of the study, this increase would not be deemed clinically relevant as all values remained within the reference range (mean %N week 5 at baseline was 2.2 ± 0.98 compared with a mean %N in week 1 of 1.3 ± 0.57, in week 3 of 1.4 ± 0.86, and in week 7 of 1.2 ± 0.72).

The reason for the observed increase in neutrophil percentage at this time point is unknown. The weather was stable, there were no differences in feed batch, and there was no known exposure to aeroallergens, although the latter would seem a logical possible explanation. A general carry-over effect is considered less likely as all other data indicate the length of the washout period seemed to be appropriate.

We cannot explain the significantly higher mast cell percentage in the first week compared with weeks 3 and 5, meaning we cannot rule out this may have been due to unmeasured environmental factors for which we could not control.

#### 4.3.6. Aspiration Techniques

A study performed by Bowser et al. (2018) evaluated the effect of manual (syringe) aspiration versus mechanical suction [30], showing a larger volume of BAL fluid recovered and a lower total red blood cell (RBC) count after manual aspiration compared with mechanical suction [31]. Corroborating this, erythrocytes were not detected in BAL fluid in our study, suggesting that the BAL procedure with manual aspiration is safe. Also, the airway mucosa where the endoscope was wedged showed only slight erythema after the baseline BAL. An unintended practical advantage of the slight mucosal color change was that the previous sample locations were identifiable at T = 48 h, making the procedure easier to perform.

## 5. Conclusions

This experimental study in four healthy horses showed a significant increase in neutrophil percentage 48 h after BAL, which was irrespective of lung lobe sampled or type of lavage solution used. Our findings suggest that fluids with a pH close to 7.4 rather than 5.5 (saline) do not reduce the neutrophilic influx subsequent to BAL in horses, supporting the current common use of saline for this procedure. No differences in BAL cytology were detected among the different lung lobes, and the effect of the BAL procedure on neutrophil influx was no longer apparent after 12 days. For future research purposes, this study also suggests that BAL procedures can be safely performed simultaneously in up to four different lung lobes in healthy horses.

## Data Availability

The data that support the findings of this study are available from the corresponding author upon reasonable request.

## Supplementary Materials

The following supporting information can be downloaded at: https://www.mdpi.com/article/10.3390/ani13162637/s1, Supplement: All data.
