# Supplementary material for: Effect of Lavage Solution Type on Bronchoalveolar Lavage Fluid Cytology in Clinically Healthy Horses
Source: Animals (Basel). 2023 Aug 15;13(16):2637. doi: 10.3390/ani13162637 (PMC10451886; doi:10.3390/ani13162637)
Supplement: Supplementary file 1 [file animals-13-02637-s001.zip › animals-2532452-supplementary.pdf]

SUPPLEMENT

all data

Fluid	Horse	Lung lobe	TNC* T=0	TNC* T=48hr	%N T=0	%N T=48hr	%M T=0	%M T=48hr
**Saline**	1	RAcL	274	803	.3	58.1	6.5	1.0
**Saline**	1	RCaL	415	478	.3	25.8	1.5	.6
**Saline**	1	LCaL	731	376	1.2	4.9	2.5	1.7
**Saline**	1	LCrL	340	282	.7	3.5	2.4	1.7
**Saline**	2	RAcL	474	781	.7	23.5	7.6	4.2
**Saline**	2	RCaL	496	626	1.4	27.3	6.4	1.7
**Saline**	2	LCaL	389	525	.5	9.9	1.3	2.2
**Saline**	2	LCrL	409	595	1.7	2.8	6.4	3.6
**Saline**	3	RAcL	828	515	1.4	6.4	1.2	1.3
**Saline**	3	RCaL	275	368	.5	12.3	1.0	.2
**Saline**	3	LCaL	594	1073	1.7	21.0	2.7	3.6
**Saline**	3	LCrL	597	488	1.3	9.5	.9	1.0
**Saline**	4	RAcL	395	372	.8	12.9	1.4	2.0
**Saline**	4	RCaL	479	873	3.3	12.3	1.0	.9
**Saline**	4	LCaL	413	433	1.8	11.4	3.1	.3
**Saline**	4	LCrL	381	684	1.3	16.2	7.2	3.4

**Ringer**	1	RAcL	506	496	.0	17.0	3.0	1.1
**Ringer**	1	RCaL	537	734	1.1	36.6	3.4	1.5
**Ringer**	1	LCaL	562	454	.9	5.5	2.9	1.3
**Ringer**	1	LCrL	337	472	1.4	5.8	2.0	1.6
**Ringer**	2	RAcL	379	554	3.6	13.1	6.0	4.1
**Ringer**	2	RCaL	476	519	3.2	25.5	1.2	1.5
**Ringer**	2	LCaL	593	1028	1.9	33.3	8.0	2.8
**Ringer**	2	LCrL	426	587	1.2	9.0	4.0	2.6
**Ringer**	3	RAcL	497	602	1.1	1.5	1.1	.7
**Ringer**	3	RCaL	540	498	1.5	12.2	.9	1.1
**Ringer**	3	LCaL	712	396	1.8	14.3	1.1	1.1
**Ringer**	3	LCrL	809	64	1.1	28.7	5.3	1.4
**Ringer**	4	RAcL	737	563	.3	2.1	6.4	4.2
**Ringer**	4	RCaL	410	464	1.2	12.0	1.5	.6
**Ringer**	4	LCaL	407	767	3.0	4.2	1.7	3.0
**Ringer**	4	LCrL	320	361	1.9	1.4	2.9	1.8
**PBS**	1	RAcL	319	456	4.3	11.0	3.1	2.1
**PBS**	1	RCaL	610	375	.8	12.4	1.4	1.6
**PBS**	1	LCaL	389	453	2.1	25.2	2.3	1.3
**PBS**	1	LCrL	368	365	.6	7.5	2.3	2.8

**PBS**	2	RAcL	291	581	1.1	8.1	4.4	4.4
**PBS**	2	RCaL	332	534	2.3	13.3	3.4	3.2
**PBS**	2	LCaL	295	323	2.4	2.6	3.5	1.9
**PBS**	2	LCrL	421	734	2.4	38.9	4.3	3.2
**PBS**	3	RAcL	485	436	1.5	12.1	2.7	2.3
**PBS**	3	RCaL	434	514	.6	12.2	1.0	1.1
**PBS**	3	LCaL	644	580	1.4	8.8	1.1	.7
**PBS**	3	LCrL	496	392	2.1	18.2	1.3	1.2
**PBS**	4	RAcL	509	709	2.0	2.6	3.7	4.4
**PBS**	4	RCaL	477	398	1.7	19.9	4.0	2.8
**PBS**	4	LCaL	481	422	2.4	7.1	.9	2.8
**PBS**	4	LCrL	402	421	2.3	6.3	1.6	3.1
**Plasmalyte**	1	RAcL	284	339	1.0	15.9	3.4	2.7
**Plasmalyte**	1	RCaL	285	218	.7	7.6	2.0	3.0
**Plasmalyte**	1	LCaL	450	496	.9	9.9	2.1	2.1
**Plasmalyte**	1	LCrL	339	219	.7	18.6	9.0	3.1
**Plasmalyte**	2	RAcL	205	588	1.0	21.3	13.3	5.6
**Plasmalyte**	2	RCaL	140	270	3.2	2.8	2.8	3.7
**Plasmalyte**	2	LCaL	227	339	3.2	1.2	7.6	5.7
**Plasmalyte**	2	LCrL	228	313	2.1	11.0	4.4	3.8

**Plasmalyte**	3	RAcL	540	385	.3	13.2	.0	.3
**Plasmalyte**	3	RCaL	446	296	1.2	18.3	6.3	4.2
**Plasmalyte**	3	LCaL	291	225	1.2	1.5	3.2	3.7
**Plasmalyte**	3	LCrL	374	330	1.8	7.2	1.3	1.3
**Plasmalyte**	4	RAcL	266	333	2.9	4.7	1.3	3.2
**Plasmalyte**	4	RCaL	308	408	1.1	11.2	3.0	1.2
**Plasmalyte**	4	LCaL	865	330	1.8	6.6	5.2	4.8
**Plasmalyte**	4	LCrL	290	296	1.9	9.2	2.2	4.3

*TNC Total nucleated cell counts (nx10^6)

%N Neutrophil percentage

RAcL Right accessory lobe

RCaL Right caudodorsal lobe

LCaL Left caudodorsal lobe

LCrL Left cranial lobe

PBS Phosphate buffered saline

TNC Total nucleated cell counts
